# Comparison between logistic regression and neural networks to predict death in patients with suspected sepsis in the emergency room

**DOI:** 10.1186/cc3054

**Published:** 2005-02-17

**Authors:** Fabián Jaimes, Jorge Farbiarz, Diego Alvarez, Carlos Martínez

**Affiliations:** 1 Associate Professor, Department of Internal Medicine and Escuela de Investigaciones Médicas Aplicadas (EIMA – GRAEPI), School of Medicine, Universidad de Antioquia, Medellín, Colombia; 2 Chairman, Department of Physiology, Universidad de Antioquia, Medellín, Colombia; 3 Assistant Professor, Department of Physiology, Universidad de Antioquia, Medellín, Colombia; 4 Assistant Physician, Department of Internal Medicine, Division of Pulmonary and Critical Care Medicine, Fundación Santa Fe de Bogotá, Bogotá, Colombia

## Abstract

**Introduction:**

Neural networks are new methodological tools based on nonlinear models. They appear to be better at prediction and classification in biological systems than do traditional strategies such as logistic regression. This paper provides a practical example that contrasts both approaches within the setting of suspected sepsis in the emergency room.

**Methods:**

The study population comprised patients with suspected bacterial infection as their main diagnosis for admission to the emergency room at two University-based hospitals. Mortality within the first 28 days from admission was predicted using logistic regression with the following variables: age, immunosuppressive systemic disease, general systemic disease, Shock Index, temperature, respiratory rate, Glasgow Coma Scale score, leucocyte counts, platelet counts and creatinine. Also, with the same input and output variables, a probabilistic neural network was trained with an adaptive genetic algorithm. The network had three neurone layers: 10 neurones in the input layer, 368 in the hidden layer and two in the output layer. Calibration was measured using the Hosmer-Lemeshow goodness-of-fit test and discrimination was determined using receiver operating characteristic curves.

**Results:**

A total of 533 patients were recruited and overall 28-day mortality was 19%. The factors chosen by logistic regression (with their score in parentheses) were as follows: immunosuppressive systemic disease or general systemic disease (2), respiratory rate 24–33 breaths/min (1), respiratory rate ≥ 34 breaths/min (3), Glasgow Come Scale score ≤12 (3), Shock Index ≥ 1.5 (2) and temperature <38°C (2). The network included all variables and there were no significant differences in predictive ability between the approaches. The areas under the receiver operating characteristic curves were 0.7517 and 0.8782 for the logistic model and the neural network, respectively (*P *= 0.037).

**Conclusion:**

A predictive model would be an extremely useful tool in the setting of suspected sepsis in the emergency room. It could serve both as a guideline in medical decision-making and as a simple way to select or stratify patients in clinical research. Our proposed model and the specific development method – either logistic regression or neural networks – must be evaluated and validated in an independent population.

## Introduction

Sepsis is the second leading cause of death among patients in noncoronary intensive care units (ICUs) and is the 10th leading cause of death overall in the USA [1]. Despite new and complex therapies, the incidence of sepsis has increased annually at a constant rate over the past 20 years, and there have been no substantial changes in the associated mortality [2].

A tool that could stratify the severity of sepsis from the initial stages in the clinical course would enhance our understanding of this disorder and its management. A simple system designed to estimate the probability of death would represent the basis for improved diagnosis, prognostication and treatment. Specifically, such a model, in the setting of the emergency room (ER), could guide decisions regarding ICU admission or whether a particular type of therapy should be instituted. The strategy may be developed from the definitions proposed by the American College of Chest Physicians/Society of Critical Care Medicine in 1992 [3]. These definitions include a generalized process with clinical findings that may represent an initial phase during the sepsis phenomenon – the systemic inflammatory response syndrome (SIRS). Although the natural history seems to reflect a continuum through different stages of an inflammatory response, from SIRS to septic shock [4], an unequivocal linear sequence of events is far from clinically apparent. Thus, classical analytical models, such as logistic regression, are limited in terms of their ability to elucidate the interplay that underlies the sepsis phenomenon.

Advances in statistical methods have supplied the tools necessary to model complex nonlinear relationships among many variables relevant to biological systems. Artificial neural networks (ANNs) are computer programs that simulate some of the higher level functions of the human brain. As in the brain, there are neurones and synapses, with various synaptic connection strengths – called 'weights' – for each connected pair of neurones. However, unlike the brain but similar to many computer programs, there is a specific set of input and output neurones for each problem and each net. These input and output neurones correspond to the inputs to and outputs from a traditional computer program. The other, termed 'hidden' neurones, along with the synapses and weights, correspond to the instructions in a traditional program. Use of ANNs as clinical prediction models has been explored in many areas of medicine, including nephrology [5], microbiology [6], radiology [7] and neurology [8]. Thus far, however, we are unaware of their use in sepsis. In this study we present a practical example that contrasts the abilities of logistic regression and neural networks to predict death in patients admitted to the ER with suspected sepsis as their main cause of hospitalization.

## Materials and methods

### Study design

In this longitudinal cohort study, patients were recruited between August 1998 and March 1999. Starting from admission to the ER, the patients were followed for 28 days or until death.

### Setting

The patients were admitted to the ERs of two reference hospitals: the Hospital Universitario San Vicente de Paúl and the Hospital General de Medellín. Hospital Universitario San Vicente de Paúl is a 550-bed, fourth level university hospital, and is a referral centre for a region including approximately 3 million habitants. Hospital General de Medellín is a 300-bed, third level teaching hospital, and is a referral centre for the metropolitan area. Both are located in Medellín, Colombia.

### Participants

We included patients aged 15 years or older with any suspected or confirmed bacterial infection as their admission diagnosis and at least one of the following SIRS criteria: temperature >38°C or <36°C; and leucocyte count >12000/mm^3^, <4000/mm^3^, or >10% immature forms (bands). We excluded eligible participants if they, their relatives, or their doctors refused to provide consent to participate in the study, or if they died or were discharged before 24 hours. Ethics committees of both hospitals had previously approved the protocol, and patients or their legal representatives signed an informed consent form.

### Measurements

The primary outcome variable was mortality within the first 28 days after admission to the ER. For those patients who were discharged before day 28, an evaluation of their vital status was conducted in the outpatient control centre or by phone if a personal interview was not possible. Independent variables recorded at admission were as follows: age, immunosuppressive systemic disease (ISD; i.e. any of cancer, chemotherapy, steroid use or AIDS), general systemic disease (GSD; i.e. any of cardiac failure, diabetes, renal failure, chronic obstructive lung disease, or cirrhosis), Shock Index (heart rate/systolic arterial pressure), body temperature, respiratory rate, Glasgow Coma Scale (GCS) score, leucocyte count, platelet count and creatinine blood level. Research assistants in the ER collected clinical variables at admission in a standardized manner. Laboratory variables were analyzed using standard quality control procedures at the participating institutions. Missing data for continuous variables were estimated with simple imputations using the median nonmissing value. In total, estimation procedures were performed in 2.6% (14 simple records) of baseline values.

### Data analysis and management

The procedure for the logistic model has been described in detail elsewhere [9]. Briefly, we conducted univariate logistic regression analysis for each candidate variable, with *P *< 0.25 being the criterion for acceptance in the model. Collinearity was checked with a matrix of correlations, using the Spearman rank correlation coefficient between independent variables. We chose a conservative strategy, with r ≥ 0.4 in at least one correlation as the criterion for multicollinearity. Logistic model assumptions (i.e. no interaction terms and a linear relationship between the logit and the continuous covariates) were verified. Then, a logistic regression analysis, employing a forward stepwise inclusion method, was developed using a *P *value of 0.05 at entry. This automatic procedure was contrasted with a backward elimination method and with a full model that included all of the candidate variables, in order to confirm the validity and stability of our results. For continuous variables, the cutoff points for changes in the probability of death were explored with locally weighted regression analysis and the lowess procedure [10]. According to the cutoff points detected, dummy variables were constructed and a new logistic regression model was fitted with those variables. In order to obtain the simplest score with the same scale within and between ranges of physiological variables and co-morbid conditions, the regression coefficients were all divided by the lowest one, and then rounded off to the nearest whole number, as the weight reflecting 'risk' for death for each variable. In defining the severity levels by the size of the coefficients, comparable severity levels within variables or conditions were grouped together. The global score for every patient in the cohort was calculated and a new logistic regression equation with the score as independent variable was fitted.

The model calibration – observed mortality versus that predicted with the score – was evaluated using the Hosmer-Lemeshow goodness-of-fit test. The test result, under a χ^2 ^distribution, provides a *P *value in which higher values (*P *> 0.05) indicate nonsignificant differences between observed and predicted mortality. The discriminatory ability – the capacity of the model to separate survivors from nonsurvivors, with 1.0 and 0.5 meaning perfect and random discrimination, respectively – was determined using receiver operating characteristic (ROC) curve analysis. Internal validation was done with 2000 bootstrap replications of the model. All statistical analyses were performed with Stata Statistical Software, Release 7.0 (Stata Corporation, College Station, TX, USA).

Using the same input and output variables, a probabilistic neural network was trained using an adaptive genetic algorithm (NeuroShell^©^; Ward Systems Group Inc., Frederick, MD, USA). The network has three neurone layers, with 10 neurones in the input layer, 368 in the hidden layer and two in the output layer, the latter indicating death versus survival. Of the cohort 75% was used to train the network and 25% was used in testing. The training criterion was that 20 generations had elapsed without changes in the minimum error. The general performance of the neural network was evaluated using the ROC curve and the Hosmer-Lemeshow goodness-of-fit test. The difference between the two ROC curves – logistic regression and neural network – was tested using the Wilcoxon statistic based on pairwise comparisons [11].

## Results

A total of 542 potentially eligible participants were admitted during the study period. Nine were excluded because of death (*n *= 5) or discharge (*n *= 4) during the first 24 hours. The final study population therefore included 533 patients, 55% (*n *= 293) of whom were male. Their age (mean ± standard deviation) was 48 ± 21 years, and their median hospital stay was 8 days (interquartile range 4–15 days). Overall 28-day mortality was 19% (*n *= 101), and 14% (*n *= 75) of the cohort was admitted to ICU.

The most common diagnoses suspected at admission were community-acquired pneumonia (recorded in 36% of patients), followed by soft tissue infection (17%), intra-abdominal infection (12%), urinary tract infection (11%) and others (11%); sepsis of undetermined source was recorded in 13% patients. The major pre-existing conditions related to admission were trauma or surgery more than 24 hours before admission (21%), chronic obstructive pulmonary disease (12%), diabetes (13%) and miscellaneous others (9%). Of the patients, 45% were free of associated diseases.

A total of 283 (53%) out of 533 cases of clinically suspected bacterial infection were microbiologically confirmed, 113 of which (40%) grew on blood samples. The rate of positive blood cultures among the total requested was 27%, and the most frequently isolated micro-organisms were *Escherichia coli *(19%), *Staphylococcus aureus *(16%), *Streptococcus pneumoniae *(13%), *Staphylococcus *coagulase negative (13%), *Klebsiella pneumoniae *(9%), *Enterobacter *spp. (6%), *Enterococcus *spp. (4%), *Streptococcus pyogenes *(3%), nonfermenting Gram-negative bacilli (3%) and others (14%).

After conducting univariate analysis for the logistic regression, leucocyte count was considered ineligible for inclusion in the model (*P *= 0.893). The evaluation of collinearity was carried out for all variables using the Spearman correlation coefficient. A significant correlation (r = 0.44) was found between age and GSD (*P *= 0.0000). Similar correlations, but to a lesser degree, were found between age and Shock Index (r = 0.1453; *P *= 0.0008) and between age and temperature (r = 0.1940; *P *= 0.0000). Therefore, age was excluded from the predictor variables. A multiple logistic regression model was applied to the overall 28-day mortality, taking into account GSD, ISD, Shock Index, respiratory rate, temperature, GCS score, creatinine and platelet count as predictive variables. This model allowed us to discard the latter two variables because they were statistically nonsignificant. For the variables respiratory rate, temperature, Shock Index and GCS score, the cutoff points for changes in the probability of death were sought by locally weighted regression. The results are shown in Table 1.

With the previous values, 12 dummy variables were constructed considering the first level (1) as the reference value. These new variables, in conjunction with the two nominal variables previously involved (GSD and ISD), were fitted in a new logistic regression model for prediction of mortality. After dividing and rounding off coefficients to the nearest whole number, some levels and variables were bound together, namely co-morbid conditions, GCS score, Shock Index and body temperature. The final meaningful variables are summarized in Table 2 according to their levels and relative weights.

In this way the final scale of severity was a range between 0 and 12. With these data, the score for each patient in the cohort was calculated, and a model that provides an estimate of severity, defined as the probability of 28-day mortality, was obtained. The Hosmer-Lemeshow goodness-of-fit test yielded a value of 7.54 (*P *= 0.5807). By ROC curve analysis for discriminative capacity, the area under the curve was 0.7517. The bootstrapped coefficients for 2000 replications exhibited standard errors of under 10% of those observed in the model, and the values for the Hosmer-Lemeshow goodness-of-fit test and the area under the ROC curve in this set were 8.96 (*P *= 0.4321) and 0.7119, respectively.

**Figure 1:**
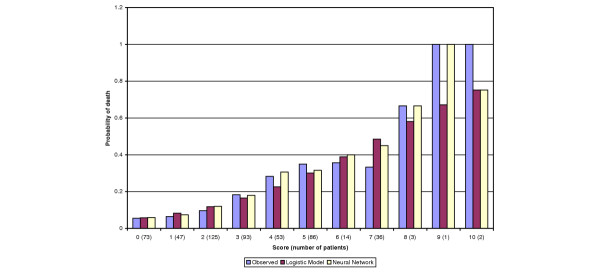
Observed and predicted deaths with logistic regression and neural network in patients with suspected sepsis admitted to the emergency room. There were no patients with scores 11 or 12 in the cohort.

The neural network included all of the independent variables. Their weight, by the smoothing factor, ranged from 2.65 for temperature to 0.34 for ISD. The Hosmer-Lemeshow goodness-of-fit test yielded a value of 8.03 (*P *= 0.475), and the area under the ROC curve was 0.8782. The difference between ROC curves was statistically significant according to the Wilcoxon statistic based on pairwise comparisons (*P *= 0.037). Figure 1 shows the comparison of observed and predicted deaths with both methods.

## Discussion

The present study shows that it is possible to obtain a simple indicator of the risk for death under clinical conditions compatible with severe infections. The system uses variables taken from the initial clinical interview and physical examination, all of which are available at the moment of admission to the ER. This suggests that it is possible to develop a reproducible and transportable predictive instrument in patients with signs indicative of sepsis. However, the model must be specifically tested in an independent population with a larger sample size. The main determinants of mortality reflect two acknowledged host factors, namely co-morbid conditions and the type of individual biological response, the latter being determined from clinical findings such as vital signs and GCS score.

The use of ANNs in the setting of sepsis has not been explored. However, with regard to overall mortality in ICUs, two recent studies compared hospital outcome prediction using neural networks versus logistic regression [12,13]. Clermont and coworkers [12] designed a prospective cohort study including 1647 patients admitted to seven ICUs at a tertiary care centre. The predictor variables considered were age and the acute physiology variables of the Acute Physiology and Chronic Health Evaluation (APACHE) III score. They constructed logistic regression and ANN models for a random set of 1200 admissions (development set), and used the remaining 447 admissions as the validation set. Then, model construction was repeated on progressively smaller development sets (800, 400 and 200 admissions) and re-tested in the original validation set. As the size of the development set sample decreased, the performance of the model on the validation set deteriorated rapidly, although the ANNs retained marginally better fit than logistic regression, as measured using the Hosmer-Lemeshow test, at 800 admissions. At under 800 admissions, however, the fit was poor with both approaches. The authors concluded that both ANN and logistic regression have similar performance with appropriate sample size, and share the same limitations with development sets on small samples.

Nimgaonkar and coworkers [13] compared the performance of the APACHE II score with that of a neural network in a medical-neurological ICU at a university hospital in Mumbai, India. A total of 2062 consecutive admissions between 1996 and 1998 were evaluated. Data from 2962 patients were used to train the neural network and data from the remaining 1000 patients were used to test the model and compare it with the APACHE II score. There were 337 deaths in these 1000 patients; APACHE II predicted 246 deaths whereas the neural network predicted 336 deaths. Calibration, as assessed using the Hosmer-Lemeshow statistic, was better with the neural network than with APACHE II score, and so was discrimination. As probable explanations for this apparent superiority of the ANN, the authors suggested differences in demographic characteristics and case-mix of patients in Indian ICUs. These specific features were certainly not accounted for in the original Western cohorts used to develop and validate the APACHE score.

In our research, both logistic regression and neural network models did a good job of predicting death. Although there was a statistically significant difference in discrimination as measured by ROC curve in favour of the neural network, the clinical meaning of this difference is not clear. A prediction model cannot be both perfectly reliable (i.e. calibrated) and perfectly discriminatory. According to Diamond [14], 'A model that maximizes discrimination does so at the expense of reliability ... On the other hand, a model that maximizes reliability does so at the expense of discrimination, and thereby trades categorical confidence for quantitative meaning.'

One of the advantages of neural network analysis is that there are few assumptions that must be verified before the models can be constructed; also, ANNs are able to model complex nonlinear relationships between independent and dependent variables, and so they allow the inclusion of a large number of variables. The comparison method is supposed to constrain the neural network analysis by limiting the number of potential predictor variables to the same set of predictor variables used in the logistic regression analysis. However, in this practical example, our network was able to use all of the 10 initial variables in its modelling, whereas logistic regression excluded four variables in the final model. Nevertheless, the predictive ability was almost the same with both approaches. A limitation of ANNs in the setting of aetiological research is that standardized coefficients and/or odds ratios corresponding to each variable cannot be calculated and presented as they can in regression models. This lack of interpretability at the level of individual predictors is one of the most criticized features of neural network models [15]. Furthermore, neural network models require sophisticated software, and the computer resources involved in training and testing neural networks can be substantial.

Our work has some limitations. First, the sample size – specifically the number of outcomes (101 deaths) – limit the number of potential predictor variables. As a rule of thumb, no more that 10 outcome events for each independent variable are permissible if over-fitting or under-fitting problems are to be avoided [16]. We tried to overcome this limitation by considering just those variables that are more likely to be related to mortality from a clinical point of view. However, as is usual in any observational study, residual confounding or unmeasured factors may compromise the scope or precision of the model. Second, external validity was tested neither for logistic regression nor for the ANN. Furthermore, the small sample size prevented a comprehensive split-sample validation with any strategy. Determination of the applicability and usefulness of any predictive model requires independent and external validation in a population that is intrinsically different from the development sample [17]. Therefore, both the proposed score and the neural network merit a new cohort study before any potential clinical use can be considered.

## Conclusion

A predictive model would be an extremely useful tool in the setting of suspected sepsis in the ER. It could serve both as a guideline in medical decision-making regarding ICU admission or specific therapies, and as a simple way to select or stratify patients for clinical research. Our proposed model and the specific development method – either logistic regression or neural networks – must be evaluated and validated in an independent population. Further research is required to determine whether there are practical or clinical advantages to one approach over the other. As a general concept, we agree with Tu [15] that logistic regression remains the best choice when the primary goal of model development is to examine possible causal relationships among variables, but that some form of hybrid technique incorporating the best features of both approaches might lead to the development of optimal prediction models.

## Key messages

- Simple clinical variables were useful in predicting death in patients with suspected sepsis in the ER.

- Logistic regression and ANNs were equivalent in terms of predictive ability.

- Discriminative ability, as measured using ROC curve analysis, was better with the ANN.

- Further research is required to validate the model and to determine whether there are practical or clinical advantages to one approach over the other.

## Abbreviations

ANN = artificial neural network; APACHE = Acute Physiology and Chronic Health Evaluation; ER = emergency room; GCS = Glasgow Coma Scale; GSD = general systemic disease; ICU = intensive care unit; ISD = immunosuppressive systemic disease; ROC = receiver operating characteristic; SIRS = systemic inflammatory response syndrome.

## Competing interests

The author(s) declare that they have no competing interests.

## Authors' contributions

FJ conceived the study, participated in its design and coordination, performed the statistical analysis for logistic regression, and drafted the manuscript. CM participated in the design and coordination of the study, and contributed to the statistical analysis. JF and DA participated in the design of the study and performed the procedures for the neural network analysis. All authors read and approved the final manuscript.

## Figures and Tables

**Table 1:** Cutoff points on continuous variables for changes in the probability of death according to locally weighted regression

Variable	Cutoff points		
	
	1	2	3
Respiratory rate (breaths/min)	<24	24–33	≥ 34
GCS score	>12	8–12	<8
Temperature (°C)	>38	36.6–38	≤ 36.5
Shock Index	<1	1–1.4	≥ 1.5

GCS, Glasgow Coma Scale.

**Table 2:** Level of variables and relative weight according to their score

Variable	Level of variable	Score
GSD or ISD^a^	Presence of GSD or ISD	2
Respiratory rate	Rate >34 breaths/min	3
Respiratory rate	Rate 24–33 breaths/min	1
GCS score	Score <12	3
Temperature	<38°C	2
Shock Index	≥ 1.5	2

^a^See text for definitions of general systemic disease (GSD) and immunosuppressive systemic disease (ISD). GCS, Glasgow Coma Scale.
